# Outcomes following corticosteroid injection for temporomandibular joint arthritis in juvenile idiopathis arthritis

**DOI:** 10.1186/1546-0096-9-S1-P122

**Published:** 2011-09-14

**Authors:** E Smith, M Friswell

**Affiliations:** 1 Great North Children’s Hospital, Richardson Road, Newcastle Upon Tyne, NE1 4LP, UK

## Background

Corticosteroid injection to the Temporo-Mandibular (TMJ) joint in children with Juvenile Idiopathic Arthritis (JIA) has been shown to be effective, but debate continues whether radiological guidance for needle placement produces better outcomes.

## Aim

To review clinical outcomes following TMJ injection without radiological guidance.

## Methods

All patients having TMJ injections (Triamcinolone Acetonide or Hexacetonide) in 2003-2009 were identified. Injections were performed under general anaesthesia. Notes were retrospectively reviewed looking at: symptoms, response to treatment, and examination findings (jaw deviation, jaw opening, and micrognathia).

## Results

47 patients (59% female) had a total of 71 joints injected. Pain and reduced jaw opening were the most common preceding symptoms. Mean age at JIA diagnosis was 9.3 years, and at time of TMJ injection was 12.8. Mean time between JIA diagnosis and first TMJ injection ranged from 0.9-14.5years. JIA subtypes were: oligoarticular (18.6%), extended-oligoarticular (16.5%), polyarticular (37.2%), enthesitis-related (9.3%), and psoriatic (18.6%). At the time of TMJ injection 18 patients were receiving methotrexate, 4 prednisolone, 1 etanercept and 1 leflunomide.

3 months after TMJ injection, 51% had complete resolution of symptoms, 44% had improved and 5% had no response. 36 patients had follow-up data at a mean of 2.5 years, showing complete resolution of symptoms in 29/36 (80.5%), improvement in 3/36 (8.3%) and no improvement in 4/36 (11.2%). No cases of micrognathia, Cushing’s syndrome, or subcutaneous atrophy were documented.

## Conclusions

In this retrospective series corticosteroid injection of the TMJ without radiologically-guided needle placement, was a safe and effective treatment with no adverse side-effects documented.

